# Tobacco use and cognitive impairment: A bibliometric analysis of research trends and knowledge structure

**DOI:** 10.18332/tid/226606

**Published:** 2026-08-27

**Authors:** Shengyuan Xu, Shaojie Qi

**Affiliations:** 1 Graduate School of Information Sciences, Tohoku University, Sendai, Japan; 2 Research Institute of Social Development, Southwestern University of Finance and Economics, Chengdu, China

**Keywords:** tobacco use, cognitive impairment, dementia, bibliometrics, knowledge mapping

## Abstract

**INTRODUCTION:**

Smoking is recognized as a key preventable risk factor for dementia and has therefore received extensive research attention. However, the relationship between smoking and cognitive impairment is complex, involving methodological controversies such as residual confounding, the nicotine paradox, and selective survival bias. Despite the growing body of evidence, this interdisciplinary field still lacks systematic bibliometric analysis. The purpose of this study is to use bibliometric methods to map the knowledge structure, thematic evolution, and emerging frontiers in smoking and cognitive impairment research.

**METHODS:**

Using the Web of Science core collection as the data source, 811 relevant English-language articles and reviews published from 1985 to 2026 were analyzed by bibliometrics. This article uses VOSviewer and CiteSpace to conduct co-occurrence and cluster analyses, burst detection, and thematic evolution analysis. It systematically examines the publication trends, collaboration networks, disciplinary distribution, keyword co-occurrence patterns, and research frontiers in this field.

**RESULTS:**

Neuroscience and Public Health constitute the two core hub disciplines. The United States (293 publications), China (213), and England (131) dominate global research output. Keyword co-occurrence analysis revealed a four-dimensional research structure centered on disease outcome, tobacco exposure, epidemiological association, and preventive intervention. Burst detection identifies a three-stage evolution path from disease subtyping and population characteristics to prevention and intervention through mechanism exploration and exposure expansion. Prevention (burst strength = 4.20) and Intervention (3.85) are the keywords with the highest intensity after 2020. Observational studies predominated, while evidence from interventions and participation by low- and middle-income countries remained limited.

**CONCLUSIONS:**

The research on tobacco use and cognitive impairment has shifted from risk identification toward intervention validation, but observational research still dominates, and intervention evidence remains limited. Low- and middle-income countries remain under-represented in global evidence production.

## INTRODUCTION

Cognitive impairment (including mild cognitive impairment and dementia) and its precursor stage of cognitive decline have become one of the most serious challenges in the field of global public health in the 21st century^1^. According to the World Health Organization’s global status report ‘Dementia’, 57 million people were living with dementia in 2021, with nearly 10 million new cases arising per year^1^. Facing this epidemiological trend, identifying and intervening with modifiable risk factors has become a core strategy to delay cognitive decline and reduce the burden of dementia^2,3^. Among the many modifiable lifestyle factors, tobacco use has received extensive attention due to its high prevalence, clear biological toxicity, and policy feasibility of global tobacco control. According to the WHO Tobacco Trends Report (2025), although the global smoking rate continues to decline, there are still about 1.2 billion tobacco users in 2024, and this huge absolute population base means that the public health impact of tobacco-related cognitive impairment will continue for a long time^2^. At the same time, about 14% of dementia cases worldwide can be attributed to smoking^3^. If this risk factor is eliminated, a considerable proportion of dementia cases can be avoided in theory, and its public health significance is worthy of attention.

However, the above attribution estimates are mainly based on the macro-level associations derived from observational studies. Going deep into the causal mechanism and effect heterogeneity at the individual level, the relationship between smoking and cognitive impairment becomes complicated. First, in some cohort studies, after controlling for socioeconomic status, education level, and alcohol use, the independent effect of smoking was significantly weakened or even disappeared, suggesting that residual confounding may have exaggerated the true association between the two^4-6^. In addition, nicotine has short-term cognition-enhancing effects. An early study has reported that smokers have better instantaneous performance in attention and working memory tasks than non-smokers^7^. This ‘nicotine paradox’ blurs public health information of the long-term impact of smoking on cognition^7^. Selective survival bias also deserves attention^8^. Smokers are more likely to die prematurely from cardiovascular and cerebrovascular diseases or cancer before entering the high-risk age group of cognitive decline. This competitive mortality rate may lead to structural bias in the sample of middle-aged and elderly smokers in observational studies, thereby underestimating the true harm of tobacco exposure^8^. The directions of these three types of biases are not consistent: residual confounding tends to overestimate risk, selective survival bias tends to underestimate harm, and the short-term cognitive effects of nicotine confuse public perception of long-term effects. The superposition of the three makes the accurate estimation of the net effect to be confronted by significant methodological challenges. It should be emphasized that the above controversy does not negate the harmful effects of tobacco use on cognitive health. Multiple lines of evidence from large cohort studies, Mendelian randomization studies, and toxicology experiments generally still support the causal association between the two, and the controversy focuses on the effect magnitude rather than the effect direction^9-11^.

Despite the above controversies in effect estimation, the neurobiological mechanism of cognitive impairment caused by tobacco exposure has been well-established. Tobacco smoke contains more than 7000 kinds of chemical substances, among which nicotine, polycyclic aromatic hydrocarbons, heavy metals and oxidizing gases can cross the blood–brain barrier and directly or indirectly damage the central nervous system through oxidative stress, neuroinflammation, vascular endothelial injury and β-amyloid deposition^12-14^. The harm of tobacco exposure is not limited to active smokers. Secondhand smoke and thirdhand smoke residue are also significantly associated with cognitive decline, and a decade-long latent effect was observed between childhood exposure and impaired executive function in the elderly^15,16^. Although epidemiological evidence and biological mechanisms are increasingly complete, global tobacco control policy evaluation still focuses on cardiovascular and cerebrovascular diseases and cancer, and cognitive health endpoints have not been included in the core benefit evaluation framework for tobacco control policy. According to the WHO Report on the Global Tobacco Epidemic (2025), more than 6.1 billion people were protected by the MPOWER best-practices policy in 2024, but only four countries have fully implemented all of these measures^17^. The policy framework for explicitly including dementia prevention as a core benefit indicator of tobacco control has not yet been established, and the translational gap between evidence and policy remains significant.

To bridge the translational gap, it is necessary to systematically map the knowledge structure and research landscape of this interdisciplinary field. Bibliometrics and knowledge mapping methods can quantitatively analyze the structure of research, research trends, and knowledge evolution, and provide a unique perspective for understanding the development of a specific research domain^18^. Some scholars have applied bibliometrics to tobacco-related research, revealing the distribution and evolution trend of research hotspots in this field^19^. However, bibliometric analysis focusing on the core interdisciplinary area of ‘tobacco use and cognitive impairment’ is still scarce. In view of this, and based on the Web of Science core collection database, this study employs co-occurrence analysis, cluster analysis, burst detection, and thematic evolution analysis to conduct a comprehensive knowledge mapping analysis of this interdisciplinary field. The specific research objectives include: 1) Clarify the external characteristics of the field, such as publication trends, core countries and organizations, leading authors, and the primary disciplines; 2) Reveal research hotspots and knowledge structure through keyword co-occurrence network analysis and cluster density visualization; 3) Use burst detection to identify emerging frontier directions and their evolution paths; and 4) On the basis of the above quantitative analysis, combined with the WHO global tobacco control framework, the existing research landscape on tobacco control and cognitive health policy formulation is discussed to provide a literature basis and direction reference for incorporating cognitive health endpoints into the tobacco control benefit evaluation system.

## METHODS

### Retrieval strategy

In this study, bibliometrics and knowledge mapping analysis methods were used to analyze the knowledge structure, research hotspots, and evolution trends in the field of tobacco use and cognitive impairment^20,21^. The literature data were from the Web of Science Core Collection. To cover the relevant literature as much as possible, the topic field (topics, covering title, abstract, author keywords, and keywords plus) was used for the search. The search string for cognitive impairment was: ‘dementia’ OR ‘alzheimer*’ OR ‘cognitive impair*’ OR ‘cognitive decline’ OR ‘MCI’ OR ‘mild cognitive impair*’ OR ‘memory loss*’. The search string for tobacco was: ‘smok*’ OR ‘tobacco’ OR ‘cigarette*’ OR ‘nicotin*’. Both sides of the retrieval formula are connected by the Boolean operator AND to ensure that the literature involves two dimensions at the same time. The type of literature is limited to Article or Review Article, with the language focus on English, and the time span is not set. The final search was performed on 28 March 2026, and 7398 records were initially obtained.

After deduplication in EndNote, two researchers independently performed manual screening of literature titles, abstracts, and keywords, with any disagreements resolved through discussion. Inclusion criteria were literature focused on the association between tobacco use and cognitive impairment. Clearly, they explored the effects of smoking, secondhand smoke exposure, or nicotine on cognitive function, dementia, or cognitive decline. No restrictions were imposed on the study population.

After the screening was completed, the bibliographic and citation data were exported in Full Record and Cited References formats, and a total of 811 articles were ultimately included in the analysis.

### Analytical framework and tools

The analysis tools used in this study were VOSviewer (version 1.6.20)^22^ and CiteSpace (version 7.0.R0)^23^. The specific analysis process is carried out in four progressive stages. First, annual publication statistics. By plotting the number-of-publications curve and fitting it with an exponential model, the overall growth trend and developmental stage of this field are assessed. Second, productivity distribution and cooperation network. Descriptive statistics are computed for the number of outputs by countries, organizations, and authors, and a cooperative network map is constructed in VOSviewer to intuitively illustrate the organizational characteristics of the research community. Third, the evolution of the discipline network occurs in stages. According to the inflection point of the index growth of the number of publications, the development of the field is divided into different periods. The discipline co-occurrence network is constructed for each period, and the evolution of the field from a single-discipline to multidisciplinary integration is investigated. The discipline co-occurrence network is constructed using CiteSpace; node size reflects the number of publications, and edge thickness reflects the cooccurrence intensity between disciplines. Fourth, keyword knowledge map analysis was conducted. VOSviewer is used for keyword co-occurrence network and cluster analysis to identify the main knowledge clusters. At the same time, with the help of CiteSpace’s burst detection function, the frontier hotspots emerging in different periods are identified. The analyses of the four levels above are interconnected, aiming to systematically describe the theme composition, hot-spot evolution, and frontier trends in this field.

## RESULTS

### Publication growth trends

**Figure 1:**
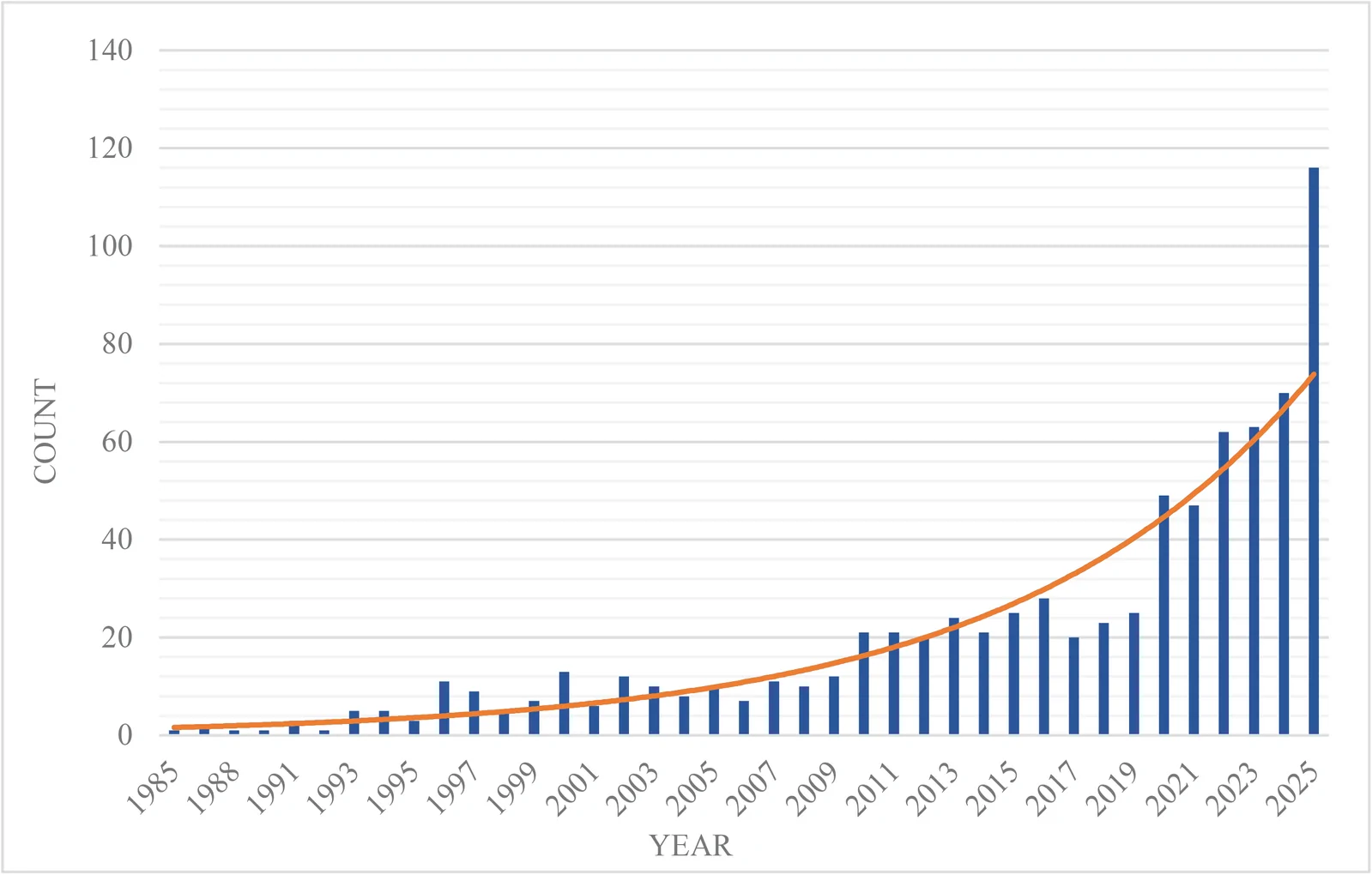
Annual publication output, 1985–2025 (N=788)

The diachronic analysis of publication numbers shows that the field has undergone a phased transition from gradual accumulation to rapid expansion. The earliest related research dates back to 1985, and the number of articles published in 1985–1995 was in the single digits. In 1996, it broke through 10 publications for the first time, reaching 11. Since then, it has been basically maintained at about 10 publications for more than ten years. Since 2010, the annual number of publications has stabilized at more than 20, and 25 in 2019. Growth accelerated significantly after 2020, jumped to 49 publications that year, and then continued to rise, reaching 116 articles by 2025 (data for 2026 were excluded from trend fitting due to the incomplete publication year). The exponential increasing function fits the trend well, with a coefficient of determination R^2^=0.92 indicating a good fit (Figure 1).

### Distribution of publication output by countries, organizations, and authors

**Figure 2:**
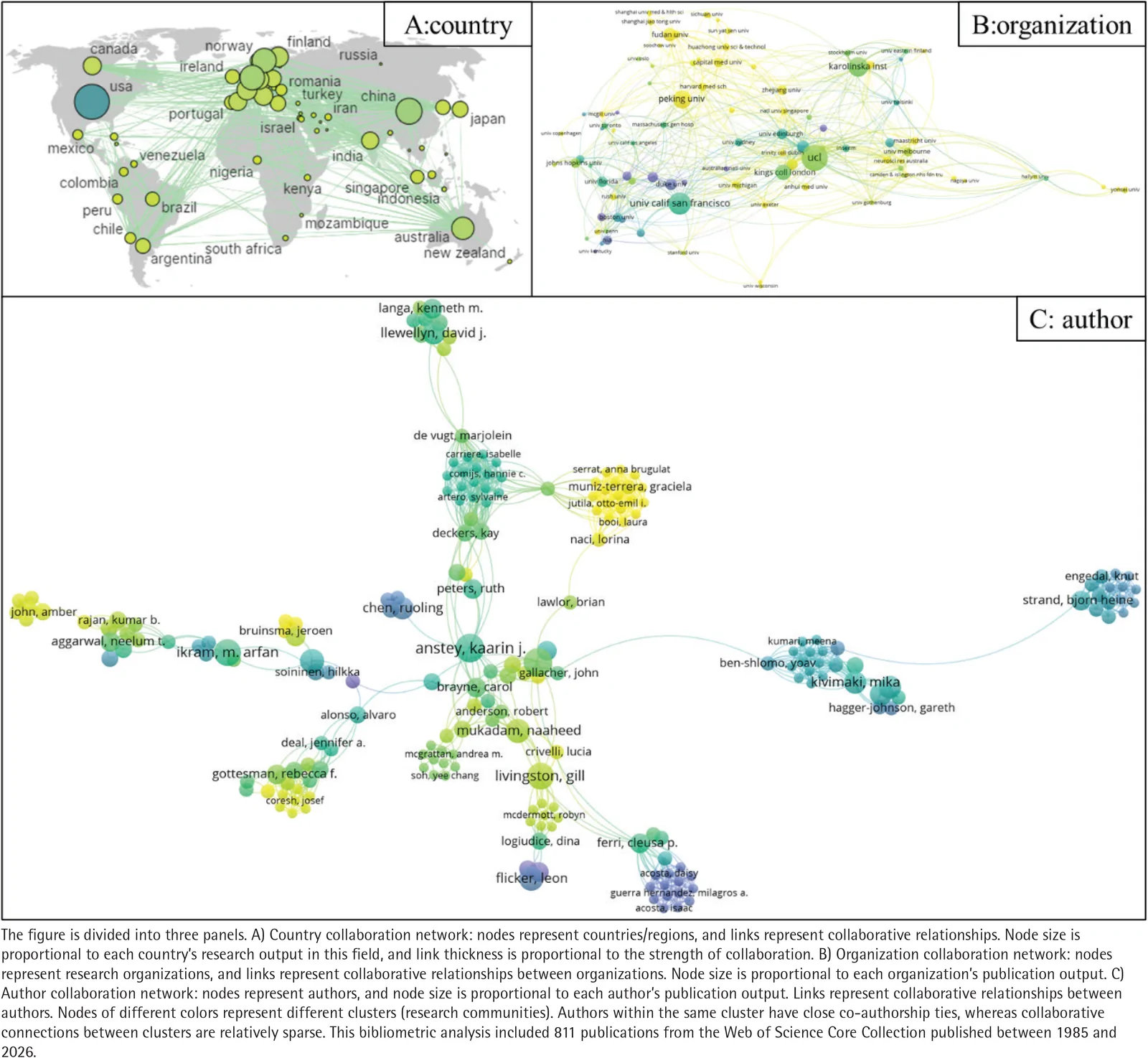
Country, organization, and author collaboration networks, 1985–2026 (N=811)

The analysis of national-level publication numbers shows that research forces in this field are highly concentrated spatially. The United States ranked first with 293 publications, China ranked second with 213 publications, followed by England (131), Australia (60) and the Netherlands (47). At the institutional level, University College London ranked first with 32 publications, followed by University of California San Francisco (27) and Peking University (22). In terms of authors, T.C. Durazzo topped the list with 10 publications, followed by K.J. Anstey, L. Tan, K. Yaffe and J.T. Yu with 8 publications each. The cooperation network map (Figure 2) further shows that the United States, China, and England constitute the hubs of global cooperation, but the overall network density is low, and the cross-border cooperation between authors remains relatively sparse (Table 1).

**Table 1:** Top 10 countries, organizations, and authors by publication count, 1985–2026

*Ranking*	*Country*	*Count*	*Organization*	*Count*	*Author*	*Count*
1	USA	293	University College London	32	T.C. Durazzo	10
2	China	213	University of California San Francisco	27	K.J. Anstey	8
3	England	131	Karolinska Institutet	24	L. Tan	8
4	Australia	60	Peking University	22	K. Yaffe	8
5	Netherlands	47	King’s College London	19	J.T. Yu	8
6	Canada	37	Fudan University	16	A. Hofman	7
7	Sweden	36	University of Cambridge	15	M.A. Ikram	7
8	Japan	28	Duke University	14	G. Livingston	7
9	South Korea	28	University of Oxford	14	D.J. Meyerhoff	7
10	France	24	Harvard University	13	L. Flicker	6

Count represents the number of publications for each country, organization, or author. This bibliometric analysis included 811 publications from the Web of Science Core Collection published between 1985 and 2026, comprising 4504 authors, 1386 organizations, and 70 countries/regions.

### Disciplinary distribution and evolution

The statistical analysis of the discipline classification reveals that the field exhibits significant interdisciplinary characteristics and that the discipline structure continues to evolve. A total of 63 Web of Science subject categories were included in the literature. The top five disciplines were: *Clinical Neurology* (199 publications, 24.54%), *Neurosciences* (195 publications, 24.04%), *Public, Environmental & Occupational Health* (128 publications, 15.78%), *Geriatrics & Gerontology* (124 publications, 15.29%), and *Psychiatry* (118 publications, 14.55%). We used the Betweenness Centrality to measure the extent to which a discipline serves as a ‘bridge’ connecting other disciplines in the co-occurrence network, with a higher value indicating a more prominent core position. The two core disciplines with the highest centrality are *Neurosciences* and *Public, Environmental & Occupational Health* (Table 2).

**Table 2:** Top 10 subject categories by frequency and betweenness centrality, 1985–2026

*Ranking*	*Count*	*Betweenness* *centrality*	*Disciplines*
1	199	0.29	Clinical Neurology
2	195	0.46	Neurosciences
3	128	0.36	Public, Environmental & Occupational Health
4	124	0.22	Geriatrics & Gerontology
5	118	0.25	Psychiatry
6	62	0.06	Medicine, General & Internal
7	57	0.16	Gerontology
8	41	0.16	Pharmacology & Pharmacy
9	37	0.00	Multidisciplinary Sciences
10	27	0.06	Substance Abuse

Count represents occurrence frequency. Betweenness centrality measures the extent to which a discipline lies on the shortest paths between other disciplines, with higher values indicating a more prominent hub position in the knowledge network. This bibliometric analysis included 811 publications from the Web of Science Core Collection published between 1985 and 2026, covering 63 subject categories.

**Figure 3:**
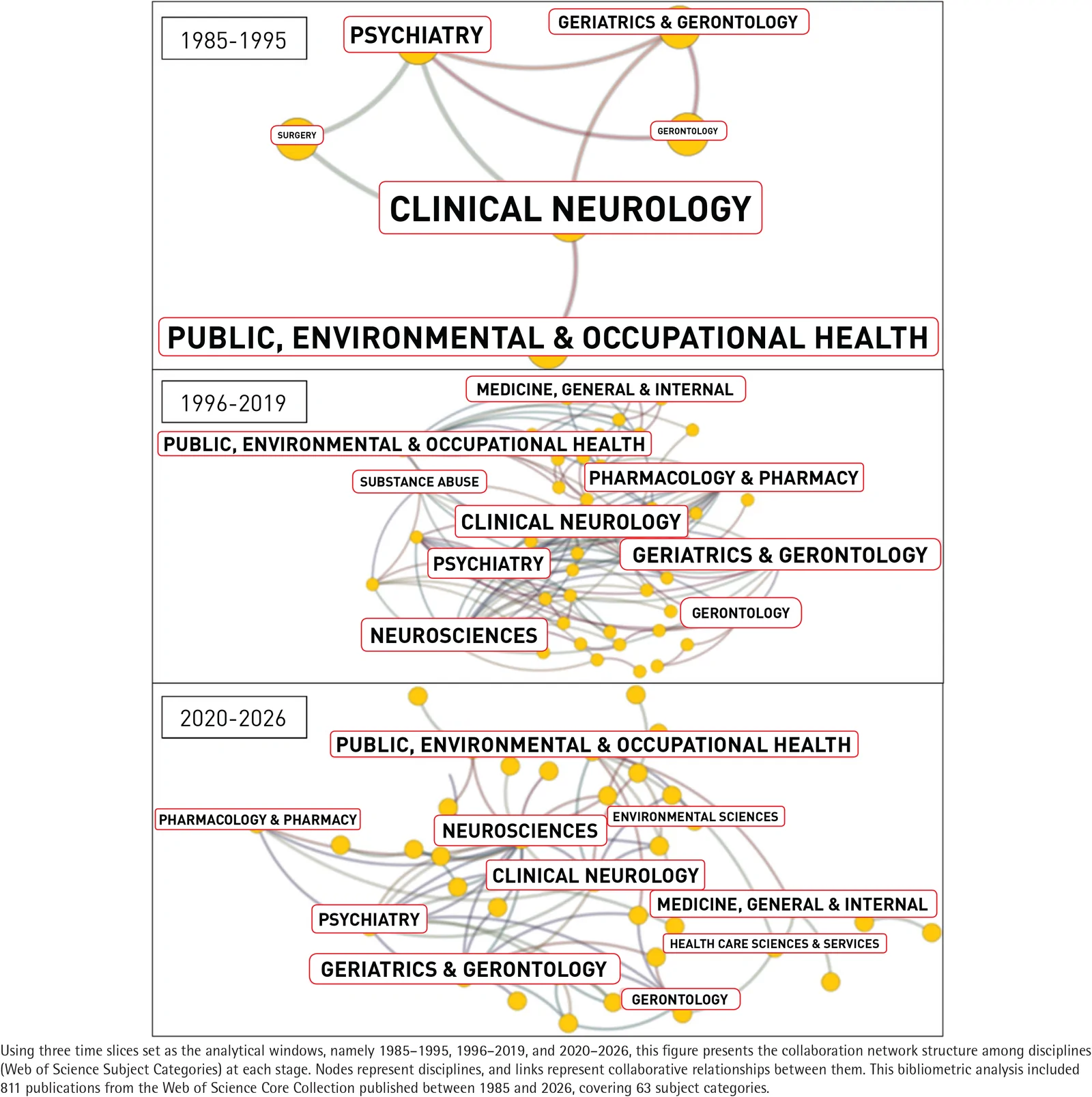
Evolution of disciplinary collaboration networks across different stages, 1985–2026

The diachronic evolution of the discipline network shows obvious stage characteristics (Figure 3). During the germination period (1985–1995), the disciplinary structure was single, and the core nodes were limited to *Public, Environmental & Occupational Health*, *Clinical Neurology*, *Psychiatry*, and *Geriatrics & Gerontology*. During the period of steady development (1996–2019), the discipline network expanded rapidly. *Neurosciences*, *Pharmacology & Pharmacy*, *Medicine* (General & Internal), and *Substance Abuse*, along with other disciplines, were integrated into the main network, significantly enhancing interdisciplinary connections. During the rapid expansion period (2020–2026, with 2026 covering only data up to the search date), the integration of disciplines has been further deepened, new nodes such as *Environmental Sciences* and *Health Care Sciences & Services* have entered, and the research pattern has become more diversified.

### Keyword co-occurrence and knowledge structure

**Figure 4:**
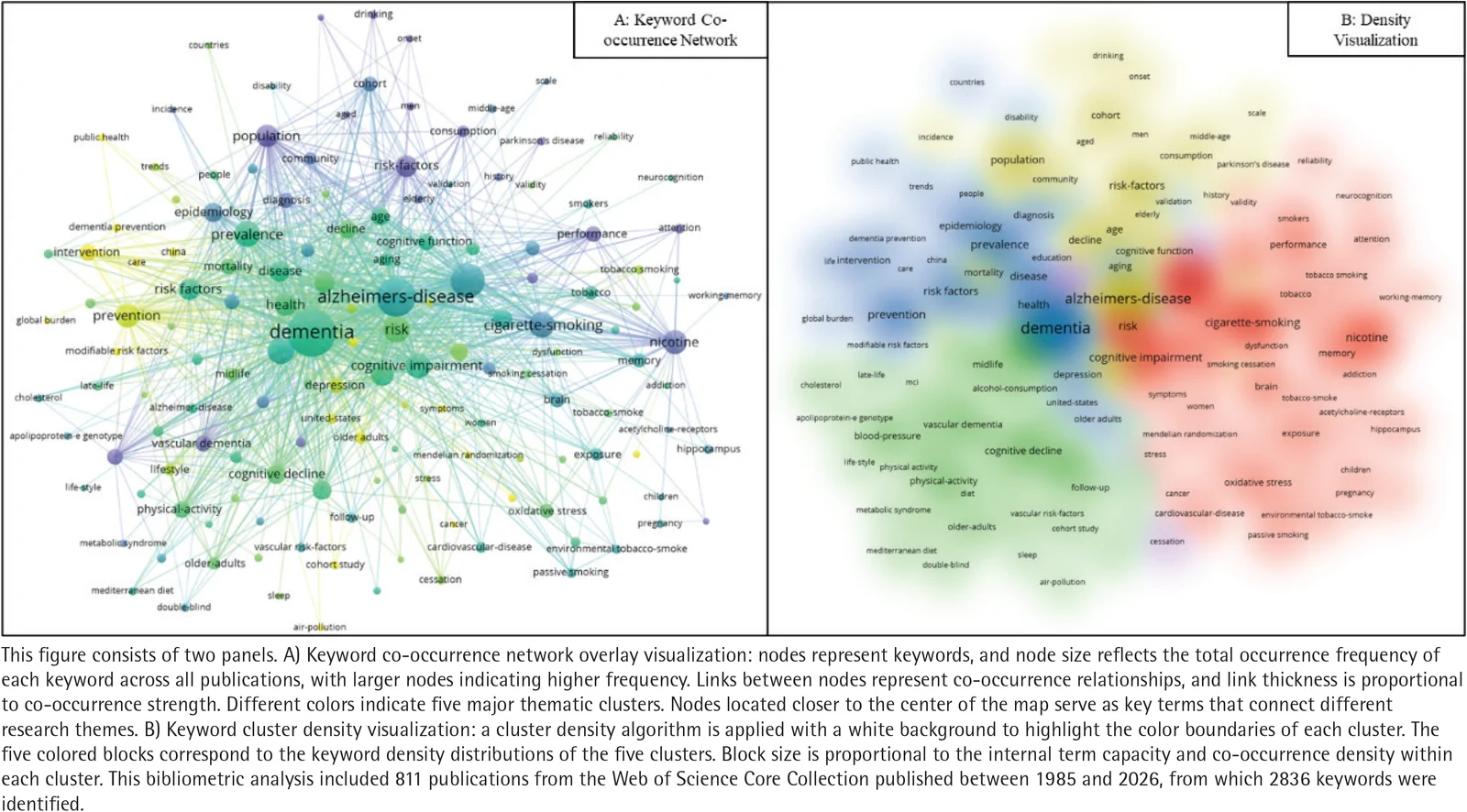
Keyword co-occurrence network map and cluster density visualization, 1985–2026

The construction of a keyword co-occurrence network provides a quantitative basis for identifying the core knowledge structure in this field. The keyword co-occurrence network is a network composed of keywords as nodes, with co-occurrence within the same publication represented by links. The size of nodes reflects the frequency of occurrence, and the link thickness reflects co-occurrence strength. A total of 2836 keywords are included. To retain the greatest diversity of topics, the frequency threshold is set to 1, meaning that all keywords that have appeared are included in the network. The high-frequency words showed that Alzheimer’s disease (the WoS standardized form of Alzheimer’s disease) and dementia were in core positions, and that the word frequencies of risk factors, association, prevalence, epidemiology, and population were high. Cognitive impairment, cognitive decline, mild cognitive impairment, and cognition were also concentrated within the network. Prevention and meta-analysis also appeared among the prominent keywords. Based on the co-occurrence characteristics of the above keywords, a four-dimensional research structure centered on disease outcomes, tobacco exposure, epidemiological associations, and preventive interventions was constructed (Figure 4).

### Research frontiers and burst evolution

The burst analysis identified 32 keyword hotspots emerging across different periods, clearly showing how the research frontier in this field gradually shifted over time. Burst detection is used to identify keywords with a sharp increase in frequency in a specific period of time, and its intensity reflects the surge in academic attention during this period^24^. The results show that in the early stage of field development, Parkinson’s disease, vascular dementia, population, education, and cohort emerged successively during the initial stage. Subsequently, blood pressure (burst strength=2.34; 2009–2014) was followed by cigarette smoking (burst strength=3.10; 2011–2017), risk factor, brain, secondhand smoke, and environmental tobacco smoke, in rapid succession. The research focus has expanded from the behavioral health effects of active smoking to the direct effects of secondhand smoke exposure and tobacco constituents on the central nervous system. After 2013, meta-analysis continued its surge, and mild cognitive impairment, oxidative stress, vascular risk factors, decline, and midlife gradually attracted growing attention.

In recent years, the frontier has increasingly focused on prevention (burst strength=4.20; 2020–2026) and intervention (burst strength=3.85; 2021–2026); they have the highest intensity of emergence after 2020; risk, age, depression, health, prevalence, physical activity, older adults and smoking have also emerged in recent years. From early to recent, the emerging themes clearly show the evolution path from disease classification and population characteristics, through mechanism exploration and exposure extension, to prevention intervention and comprehensive risk management (Supplementary file Figure 1). It is worth adding that the evolution of the above emergent themes is synchronized with the phased expansion of the discipline network at the macro level. Early burst terms are concentrated in the period dominated by the disciplines of Clinical Neurology and Geriatrics & Gerontology, while recent burst terms appear in the context of the integration of new disciplines such as Environmental Science and Health Care Sciences & Services.

## DISCUSSION

### Global cooperation landscape

Analysis at the country level showed that the United States and China were the leading contributors, followed by England. However, the overall density of the cooperation network is low, and transnational cooperation among authors is relatively dispersed. This high-output yet fragmented pattern is largely due to the field’s interdisciplinary nature.

Researchers are distributed in multiple disciplines, and there are more intra-disciplinary exchanges than interdisciplinary collaborations. At the level of organizations and authors, T.C. Durazzo, and four other authors, constitute the productive group, but direct cooperation among high-yield authors is limited, suggesting that the field has not yet formed a core author group with close collaboration.

It is worth noting that the country distribution landscape of this study shows that low- and middle-income countries (LMICs) are under-represented in this field. Research engagement in Africa, South Asia, and Latin America is low, and these regions are experiencing the world’s most rapid population aging. This evidence deficit means that almost all current evidence on the tobacco-cognitive association comes from high-income countries, and the applicability of its research conclusions in LMICs has yet to be sufficiently empirically tested^25^.

### Knowledge structure of research hotspots

The keyword co-occurrence analysis of this study identified four core research hotspots in this field: 1) epidemiological confirmation of the association between smoking and dementia risk, 2) dual neuropharmacological effects of nicotine, 3) secondhand smoke and extended exposure, and 4) prevention and intervention. There is an inherent progressive relationship among the four hotspots that outlines the evolution of the field from risk identification to intervention validation.

*Epidemiological confirmation of the association between smoking and dementia risk*

This line of inquiry has accumulated the most extensive body of knowledge. Alzheimer’s disease and dementia are at the core of the keyword co-occurrence network. The meta-analyses of Anstey et al.^4^ and Durazzo et al.^5^ established the status of smoking as a modifiable risk factor for dementia. Follow-up studies further focused on methodological issues such as the dose-response relationship^26^, the critical exposure window period^27^, and selective survival bias^8^, so that the evidence base for this association tends to be stable. The main challenge is that the interference of residual confounding on effect estimation has not been completely ruled out, and the independent risk of smoking still fluctuates under different adjustment strategies^4,6,8^.

*Dual neuropharmacological effects of nicotine*

Nicotine can improve cognitive function in the short-term, while tobacco smoke accelerates neurodegeneration through amyloid-beta (Aβ) and tau pathology^28,29^. The core issue of this debate is whether the short-term pharmacological effects of nicotine can be safely dissociated from the overall toxicity of tobacco. Because it is impossible to confirm whether the short-term cognitive benefits of nicotine will be offset by the long-term toxicity of other components in smoke, the safety of nicotine-based intervention strategies has always been questioned^30^. At present, clinical trials around α7-nAChR selective agonists are advancing, but data on long-term cognitive endpoints are still lacking^30^.

*Secondhand smoke and extended exposure*

The research scope has been extended from active smoking to passive exposure; the cognitive risk of secondhand smoke has been confirmed^15^, and the long-term effects of early life exposure have also begun to receive attention^16^. However, the neurotoxicity of thirdhand smoke is still a research blind spot – the long-term low-dose exposure risk of tobacco residues adsorbed on the surface of indoor objects to infants and the elderly at home, which has hardly been systematically explored. At the same time, the accurate quantification of exposure doses remains unresolved. Most studies still rely on self-report questionnaires, and it is difficult to capture individual differences in actual exposure levels.

*Prevention and intervention*

Prevention and intervention are the keywords with the highest burst strength in recent years, reflecting the field’s expansion from risk identification to intervention validation. However, this shift has not yet been translated into substantive intervention evidence, and randomized controlled trials of smoking cessation with cognitive protection as the end point are still almost blank. This is the most prominent weak link in the current research landscape. Whether the lower dementia risk of smokers in observational studies actually comes from the smoking cessation behavior itself, or partly reflects the health choice bias, remains unclear, and there is insufficient experimental evidence to clarify it^31^.

### Dynamic evolution of research frontiers

The burst analysis in this study identified a three-stage evolutionary path in the field: from disease subtyping and population characteristics, through mechanism exploration and exposure expansion, to prevention interventions and comprehensive risk management. This path is synchronized with the phased expansion of the discipline network at the macro level. Early burst terms are concentrated in the period dominated by clinical neurology and geriatrics, while recent burst terms appear in the context of integrating new disciplines such as environmental science and health services.

The first stage (1985–2008) was marked by the emergence of Parkinson’s disease, vascular dementia, and population and cohort studies. This stage is dominated by observational study design. The core task is to establish a case definition around different subtypes of cognitive impairment, and to confirm whether there is an association between smoking and cognitive impairment by cohort design^32,33^. The main driving force for research in this period came from the accumulation of clinical cases and the application of early epidemiological methods.

The second stage (2009–2019) was characterized by blood pressure, cigarette smoking, secondhand smoke, and environmental tobacco smoke as key terms. Research focus was extended from active smoking to secondhand smoke and the direct impact of tobacco constituents on the central nervous system^34^. At the same time, meta-analyses continue to emerge, and mild cognitive impairment, oxidative stress, vascular risk factors, and midlife have gradually attracted attention. The research landscape has shifted from confirming the existence of associations to quantifying their intensity and elucidating their underlying biological pathways. The maturation of large-scale aging cohort data and the widespread adoption of neuroimaging technology provide key methodological support for expanding research at this stage.

In the third stage (2020–present), marked by prevention and intervention, depression, physical activity, older adults, and smoking have emerged one after another, indicating that current research has included smoking in the multi-risk framework of elderly health^35^, and the focus of research is moving from ‘problem identification’ to ‘problem solving’^36^. Of course, the burst of intervention-related terms does not mean that intervention research has formed a scale. The distribution of literature types suggests that observational studies still dominate the field, while experimental intervention research remains in its nascent stage. This imbalance between observational and experimental research is one of the core deficiencies, discussed in the next section.

### Research gaps and future directions

Based on the systematic review of the above bibliometrics, the lack of intervention research is the most prominent structural shortcoming. The current evidence chain is biased towards observational epidemiological studies and preclinical mechanism research, and there is a lack of randomized controlled trials on smoking cessation with cognitive protection as the endpoint. Although prevention and intervention have recently exhibited the highest burst strength, the surge in burst detection reflects increased academic attention, which does not mean sufficient accumulation of high-quality intervention evidence. In the future, priority should be given to promoting smoking cessation interventions with cognitive outcomes as the endpoint, and to incorporating neuroimaging and blood biomarkers into the intermediate indicator design to capture the potential benefits of smoking cessation on cognitive health in a shorter follow-up period.

Another gap worthy of attention is the lack of coverage of research on elderly women, elderly people living alone, and low socioeconomic status groups. Gender differences and social stratification perspectives have not been fully included in tobacco-cognitive research. Furthermore, evidence of long-term neurological safety of e-cigarettes and heated tobacco products remains extremely limited. The cognitive risk assessment of these new and traditional non-active exposure sources together, constitutes an important direction for future research at the intersection of environmental health and neuroepidemiology.

At the policy level, based on the macro trend in evidence accumulation, the current benefit evaluation of the WHO MPOWER tobacco control framework still focuses on cardiovascular and cerebrovascular diseases and cancer^17^. By contrast, the bibliometric analysis of this study shows that the literature on cognitive health outcomes has accumulated a substantial body of work. Incorporating cognitive health endpoints into the global tobacco control benefit assessment framework helps to bridge the mismatch between evidence accumulation and policy attention. Strengthening the intervention status of tobacco control in the national dementia prevention action plan^1^, and embedding cognitive screening in the routine physical examination of middle-aged smokers are all worthy of policymakers’ attention.

### Limitations

This study has several limitations. The data source is limited to the Web of Science Core Collection, and some regional or non-English-related research may be omitted, potentially resulting in publication bias.

Bibliometric indicators focus on the study of output scale and network structure characteristics, which cannot fully reflect the scientific quality of individual research. Moreover, the identification of the frontier direction is based on the recent citation and emergence model, which are predictive in nature and need to be verified by subsequent empirical research.

## CONCLUSIONS

Based on a bibliometric analysis of the relevant literature on tobacco use and cognitive impairment, this study systematically constructed a knowledge landscape in this field. The study found that the field has entered an exponential growth phase since 2020 and has formed a dual-core discipline structure with neuroscience and public health, as well as four-dimensional research topics covering disease outcomes, tobacco exposure, epidemiological associations, and preventive interventions. Burst analysis revealed that the focus of research has gradually shifted from the early identification of disease subtypes, through mechanism exploration and exposure expansion, to the frontier direction with prevention and intervention as the core. However, intervention studies are still scarce, experimental evidence on the effect of smoking cessation on cognitive protection is almost blank, and research participation in low-and middle-income countries is also inadequate. This study can help identify the core issues and weak links in this field and provide macro-level evidence from the literature to support promoting the integration of cognitive health endpoints into the global framework for evaluating tobacco control benefits.

## Supplementary Material



## Data Availability

The data supporting this research are available from the authors on reasonable request.
