# Supplementary material for: Tobacco use and cognitive impairment: A bibliometric analysis of research trends and knowledge structure
Source: Tob Induc Dis. 2026 Aug 27;24:10.18332/tid/226606. doi: 10.18332/tid/226606 (PMC13520597; doi:10.18332/tid/226606)
Supplement: Supplementary file 1 [file TID-24-150-s1.pdf]

TID
Tobacco Induced Diseases

Supplementary file

© 2026 Xu S. and Qi S.

DOI:
10.18332/tid/226606

The content has been provided by the author(s) and has not been reviewed, verified, or endorsed by European Publishing. It may not have undergone peer review. The views, opinions, and recommendations expressed are solely those of the author(s) and do not necessarily reflect the position of European Publishing. European Publishing accepts no responsibility or liability for any consequences arising from the use of, or reliance on, this content.

Supplementary Figure 1. Keyword burst strength map, 1985–2026

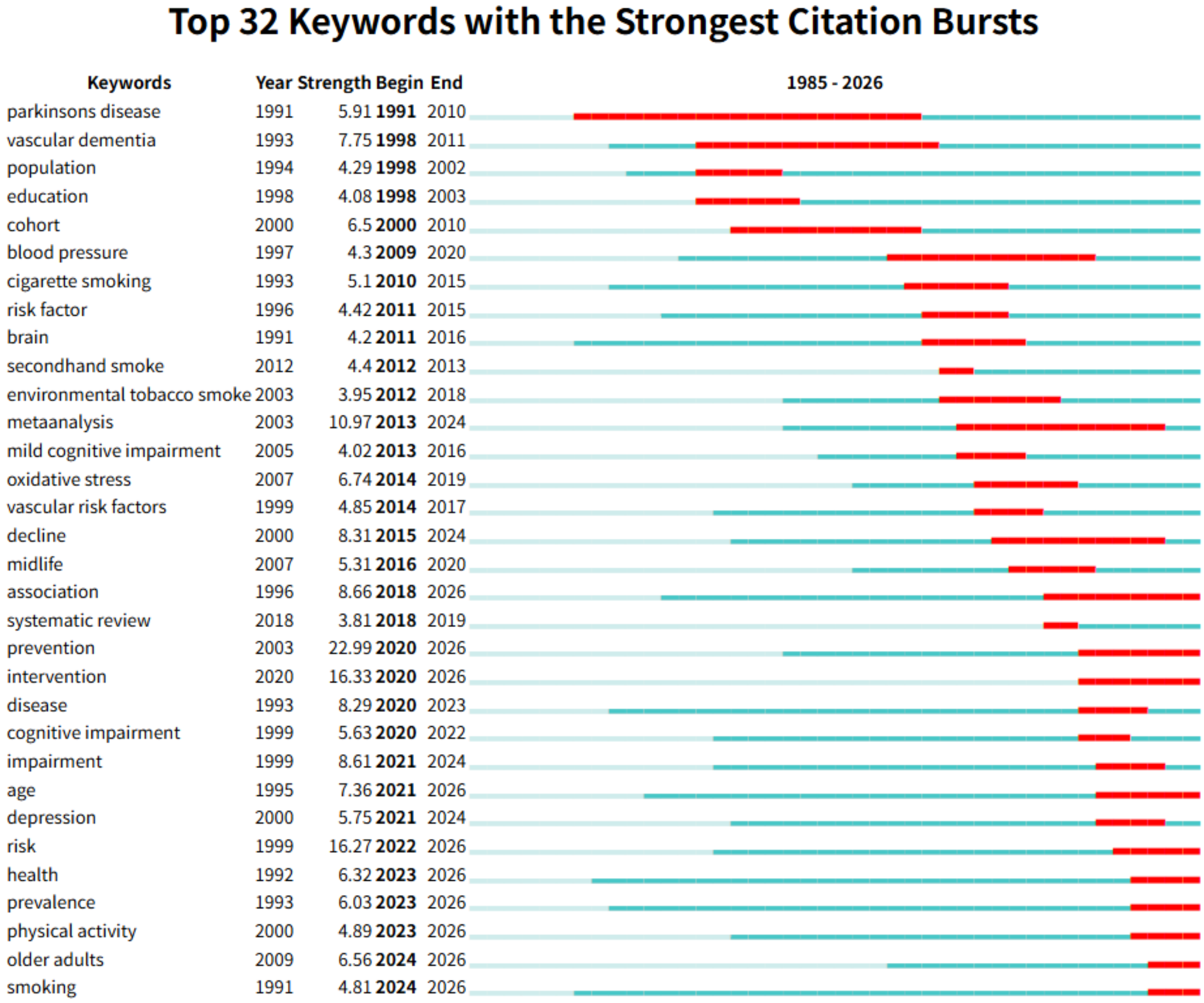

This figure displays 32 keywords with significant burst effects. Blue line segments mark the start and end years of each keyword's coverage in the literature across the entire time window, while red line segments highlight the burst-active periods during which a keyword's citation counts or occurrence frequency increased sharply. Higher burst strength values indicate a greater surge in scholarly attention to that keyword during the corresponding burst period. This bibliometric analysis included 811 publications from the Web of Science Core Collection published between 1985 and 2026.
